# Gestational Diabetes Mellitus Prevention: A Commentary

**DOI:** 10.55975/TPDP7486

**Published:** 2024-09-01

**Authors:** Catherine Gallagher, James Hill

**Affiliations:** Mersey and West Lancashire NHS Teaching Hospitals; University of Central Lancashire

## Abstract

Gestational diabetes mellitus (GDM) poses a significant global health challenge, with a prevalence ranging from 10-25%, making it one of the most prevalent complications of pregnancy. The rates vary across countries, ethnicities, and diagnostic thresholds. In the UK alone, GDM affects approximately 35,000 pregnancies annually, reflecting an escalating trend. Elevated blood glucose levels in pregnancy exhibit a linear correlation with adverse maternal and neonatal outcomes, contributing to complications such as large for gestational age babies, increased caesarean section rates, shoulder dystocia, neonatal hypoglycaemia, intensive care admissions, and stillbirth. While any woman can develop GDM, well-established risk factors include a body mass index (BMI) above 30, maternal age over 40, previous GDM instances, a history of delivering babies weighing over 4.5kg, and specific ethnic backgrounds. This commentary discusses the Cochrane umbrella review by Griffith et al. (2020), which evaluates the effectiveness of various lifestyle-focused interventions commonly employed for GDM prevention. Given the multitude of risk factors, early interventions before or during pregnancy hold promise in mitigating the likelihood of GDM development.

## Introduction

The worldwide prevalence of Gestational diabetes mellitus (GDM) varies between 10-25% and is now considered one of the most common complications of pregnancy.^1^ Rates of GDM are increasing in the UK, affecting an estimated 35,000 pregnancies per annum.^2^ There is a linear relationship between blood glucose levels and adverse maternal and neonatal outcomes^3^. Gestational diabetes mellitus can cause serious complications to both mum and baby.^4^ For mums there is an increased risk of pre-eclampsia and obstetric intervention, as well as an increased risk of future GDM and a ten-fold higher risk of developing future Type 2 diabetes (T2DM)^1^. There is also an association between GDM and maternal mental health conditions including anxiety, antenatal depression, and post-traumatic stress disorder (PTSD).^5^ Thus, this condition places a significant clinical and psychological burden on women living with GDM and their families, as well as a financial implication on the National Health Service (NHS).^4,5^ Any woman can develop GDM, but risk factors are well established^6^. Due to the presence of multiple risk factors for GDM, it is probable that interventions implemented before or early in pregnancy could decrease the likelihood of GDM development.^7^ The Cochrane umbrella review by Griffith et al (2020) discussed in this commentary aims to evaluate the effectiveness of a range of lifestyle focused interventions that are commonly used in the prevention of GDM.^7^

### Aim of commentary

This commentary aims to summarise and highlight the main findings from an umbrella review undertaken by Griffith et al (2020) and expand upon these findings in context to practice.^7^

## Methods

This Cochrane review undertook a comprehensive search of the Cochrane Database of Systematic Reviews, using key words ‘Gestational Diabetes’ or ‘GDM’, from date of inception through to 6^th^ August 2019. Search terms were used to search ‘all text’ and were not limited to ‘title, abstract or keywords’. There were no language restrictions placed on the search procedure. Only randomised control trials investigating interventions which reported GDM prevention as a primary or secondary outcome were included. There were plans to include timing of intervention (women planning a pregnancy, between pregnancies or pregnant women) but all interventions included in this review were during pregnancy. Studies were excluded if they included participants with pre-existing Type-1 diabetes or Type-2 diabetes.

Comprehensive screening, data extraction and assessment of bias, using the Risk of Bias in Systematic Reviews (ROBIS) tool, were undertaken by two independent reviewers, with arbitration by a third reviewer. An overall level of evidence quality for GDM (rate of certainty) was given using the grading of recommendations, assessment, development, and evaluation (GRADE). When this was not available, the GRADE system was used to review pooled summary statistics and risk of bias of included trials. The risk of bias of included studies was not assessed as part of the review and reported study risk was based on the review authors’ assessment. All reviews performed meta-analyses for GDM, the intended outcome. A narrative synthesis of the Cochrane systematic reviews was undertaken.

## Results

Eleven Cochrane reviews (71 trials, 23,154 women) with GDM as an outcome were included. Diet and exercise were borderline statistically significant in reducing the relative risk of developing GDM compared to usual care (RR 0.85, 95% confidence interval (CI) 0.71 to 1.01, GRADE: moderate). There was a statistically significant reduction in the relative risk of developing GDM when comparing Vitamin D supplementation (RR 0.51, 95% CI 0.27 to 0.97, GRADE: low) and ‘myo-inositol + folic acid’ (RR 0.43, 95% CI 0.29 to 0.64, GRADE: low) compared to usual care/placebo/minimal patient information. There was a non-statistically significant reduction in the relative risk of developing GDM when comparing Metformin compared to placebo in obese pregnant women (RR 0.85, 95% CI 0.61 to 1.19, GRADE: moderate).

A statistically significant reduction in the relative risk of developing GDM was observed when comparing probiotics + diet to placebo + diet (RR 0.37, 95% CI 0.15 to 0.89, GRADE: very low). Similarly, a significant reduction in relative risk was observed when comparing probiotics and diet to placebo alone (RR 0.38, 95% CI 0.16 to 0.92, GRADE: very low). There is high quality evidence that there is no difference between omega-3 fatty acid supplementation vs placebo or no omega-3 on the risk of developing GDM (RR 1.02, 95% CI 0.83 to 1.26, GRADE: High). There was also moderate quality evidence that there was no difference between universal screening and treatment for thyroid dysfunction compared to risk-based screening (RR 0.93, 95% CI 0.70 to 1.25, GRADE: moderate).

There was no evidence of difference for dietary advice alone, exercise alone, vitamin D + calcium, low molecular weight heparin, low dose aspirin, low dose aspirin + low molecular weight heparin, fractional exhaled nitrogen oxide to adjust asthma therapy, pharmacist led multidisciplinary management of maternal asthma, when compared to non-active controls (standard care, placebo or no intervention) (GRADE: low to very low-quality evidence).

There was no evidence of difference between low glycaemic index diet compared to moderate to high glycaemic index diet, vitamin D + calcium + other vitamins and minerals compared to placebo calcium + other vitamins and minerals and low dose aspirin molecular low weight heparin compared to low dose aspirin or low molecular low weight heparin (GRADE: low to very low-quality evidence).

## Commentary

As highlighted in the umbrella review, out of the 11 included systematic reviews 10 were deemed to be at low risk of bias and one was unclear. Thus, these reviews provide a comprehensive summary of the question of interest.

Examining dietary interventions, there was no evidence of difference that adherence to glycaemic index diets preventative interventions reduced the risk of GDM. It is crucial to emphasise that this estimation of effect was based upon low certainty evidence (the estimated effect may differ from the true effect). For dietary advice alone there was a borderline statistically significant reduction in risk of GDM compared to usual care. Similarly, this was based upon very low certainty evidence (the true effect is probably markedly different from the estimated effect). However, a more recent systematic review in this field has suggested that dietary interventions might lead to a decrease in the likelihood of developing GDM.^8^ Additionally, in this more recent review it was identified that dietary preventative interventions may also have additional benefits on preterm delivery, total adverse maternal outcomes and total adverse neonatal outcomes.^8^ Nevertheless, there were also issues of unexplained heterogeneity for all outcomes. A systematic review investigating potential associations with an elevated risk of developing GDM revealed that diets rich in ultra-processed foods may be linked to an increased risk of GDM.^9^ Therefore, it is still uncertain what the effect of dietary advice alone may have on reducing the risk of GDM but it appears to be an important moderating factor in reducing risk of GDM.

For specific dietary supplementation, findings from this umbrella review suggest that vitamin D may decrease the risk of developing GDM. However, this is based upon low certainty evidence. Nonetheless, the current guidelines from the NHS advocate the use of daily vitamin D supplements during pregnancy (600 IU/day of vitamin D).^10^ Beyond the potential advantages of diminishing the risk of developing GDM, there are indications that vitamin D deficiency during pregnancy could lead to adverse consequences for the unborn child, in that insufficient vitamin D levels during pregnancy may be associated with an increased risk of lower bone mineral content, enamel defects, and attention deficit hyperactivity disorder.^11^ It is important to note that vitamin D toxicity is a rare occurrence, but it can happen because of excessive over-the-counter supplementation.^12^

Regarding the supplementation of myo-inositol and folic acid, this umbrella review indicated low-quality evidence suggesting that this supplementation might reduce the risk of GDM. A recent systematic review in this domain yielded a comparable effect estimate, featuring a slightly narrower 95% confidence interval and a proposed improvement in the quality of evidence to moderate using the GRADE assessment tool.^13^ Conversely since this umbrella review has been published, the Cochrane review which the umbrella review refers to has now been updated, where they have identified seven more papers but unfortunately this has resulted in a wider confidence interval and downgrading in evidence to very low.^14^ The conflicting certainty in the available evidence poses a challenge in formulating current practice recommendations for the preventive use of myo-inositol and folic acid against the risk of GDM.

Upon reviewing exercise-alone interventions in the umbrella review, the evidence suggests with a low certainty that interventions specifically targeting exercise alone may not yield a significant difference in reducing the risk of GDM. Due to the wide range of additional benefits that exercise provides the Department of Health and Social Care advises adults to do 150 minutes of moderate intensity exercise per week and at least two strength training activities per week.^15^ This umbrella review found moderate certainty evidence that when combined, exercise and diet interventions may produce a reduction in risk of GDM compared to usual care. The current guidance from the NHS aligns with these findings, recommending that women maintain a healthy weight before pregnancy through adherence to a balanced diet and participation in regular physical activity.^16^

Although very low-grade evidence, this review found a statistically significant reduction in the relative risk of developing GDM was observed when comparing probiotics + diet, to placebo + diet. Some studies have explored the use of probiotics to potentially reduce the risk of GDM by improving glucose metabolism and insulin sensitivity in pregnant women.^17^ In a more recent Cochrane review, high certainty evidence suggests an increased risk of hypertensive disorders of pregnancy (pre-eclampsia) with probiotic use^18^, therefore, caution is warranted in recommending probiotics in pregnancy at this stage. Overall, the results are mixed, making it difficult to make any recommendations to clinical practice. Further research is required to establish a clear link between probiotics and GDM prevention.

This umbrella review reports moderate quality evidence that Metformin vs placebo, given to obese pregnant women can reduce the risk of GDM. National Institute for Health and Care Excellence (NICE) guidelines recommend Metformin is offered to women who develop GDM and are struggling to achieve blood glucose targets through lifestyle changes alone.^19^ It is also prescribed on the NHS to women who have polycystic ovary syndrome (PCOS).^20^ However, there is a lack of evidence exploring long term risks associated with Metformin in pregnancy. The NHS only offers Metformin to pregnant women after GDM has been diagnosed.^20^

Additional research in this field is imperative, particularly focusing on the effects of diet, as uncertainties persist regarding the impact of diet alone or specific diet types. Recent reviews have shown some preventive effects; however, substantial heterogeneity suggests the presence of important moderating factors that warrant further exploration. Active comparisons of different diet types are recommended to identify key moderating factors. Regarding vitamin D supplementation, there is a need for more high-quality research and a thorough assessment of moderating factors. The existing evidence base is considered to be at a high risk of bias, and notable heterogeneity between studies implies potential moderating factors such as dosage, frequency, and duration of supplementation, which may contribute to variations in effects. In the case of myo-inositol and folic acid, recent systematic reviews exhibit varying findings, leading to a wide range of certainty estimates. Further research is essential to delve into the variations in certainty estimates, as these may be attributed to slight discrepancies in inclusion criteria or systematic review methods. For probiotic supplementation, it is crucial to conduct additional high-quality independent research with a placebo comparator to thoroughly assess its effects.

*This research was partly funded by the National Institute for Health and Care Research Applied Research Collaboration North West Coast (NIHR ARC NWC). The views expressed are those of the authors and not necessarily those of the NHS, the NIHR, or the Department of Health and Social Care*.

## Reflective Questions

1. What are the key limitations of this review discussed in the commentary and what needs to be considered when applying this evidence to practice?
2. What advice can be given on lifestyle modifications to reduce the risk of developing gestational diabetes mellitus?
3. What other factors need to be considered when comparing interventions targeted at preventing the developing of gestational diabetes mellitus?

## Key Points

- A combination of both diet and exercise may reduce the relative risk of developing gestational diabetes mellitus compared to usual care (GRADE: Moderate certainty).
- There is no difference between omega 3 fatty acid supplementation vs placebo or no omega 3 supplementation on the relative risk of developing gestational diabetes mellitus (GRADE: High certainty).
- There was no evidence of difference between universal screening and treatment for thyroid dysfunction compared to risk-based screening (GRADE: Moderate certainty).

## References

[1] Sweeting A, Wong J, Murphy HR, Ross GP (2022). A Clinical Update on Gestational Diabetes Mellitus. Endocr Rev.

[2] Kusinski LC, Murphy HR, De Lucia Rolfe E, Rennie KL, Oude Griep LM, Hughes D (2020). Dietary Intervention in Pregnant Women with Gestational Diabetes; Protocol for the DiGest Randomised Controlled Trial. Nutrients.

[3] Metzger BE, Lowe LP, Dyer AR, Trimble ER, Chaovarindr U, Coustan DR (2008). Hyperglycemia and adverse pregnancy outcomes. N Engl J Med.

[4] Kc K, Shakya S, Zhang H (2015). Gestational diabetes mellitus and macrosomia: a literature review. Ann Nutr Metab.

[5] OuYang H, Chen B, Abdulrahman AM, Li L, Wu N (2021). Associations between Gestational Diabetes and Anxiety or Depression: A Systematic Review. J Diabetes Res.

[6] NHS (2022). Overview -Gestational diabetes. https://www.nhs.uk/conditions/gestational-diabetes/.

[7] Griffith RJ, Alsweiler J, Moore AE, Brown S, Middleton P, Shepherd E, Crowther CA (2020). Interventions to prevent women from developing gestational diabetes mellitus: an overview of Cochrane Reviews. Cochrane Database Syst Rev.

[8] Teede HJ, Bailey C, Moran LJ, Bahri Khomami M, Enticott J, Ranasinha S (2022). Association of Antenatal Diet and Physical Activity-Based Interventions With Gestational Weight Gain and Pregnancy Outcomes: A Systematic Review and Meta-analysis. JAMA Intern Med.

[9] Paula WO, Patriota ESO, Gonçalves VSS, Pizato N (2022). Maternal Consumption of Ultra-Processed Foods-Rich Diet and Perinatal Outcomes: A Systematic Review and Meta-Analysis. Nutrients.

[10] NHS (2023). Vitamins, supplements and nutrition in pregnancy. https://www.nhs.uk/pregnancy/keeping-well/vitamins-supplements-and-nutrition/.

[11] Paula WO, Patriota ESO, Gonçalves VSS, Pizato N (2022). Maternal Consumption of Ultra-Processed Foods-Rich Diet and Perinatal Outcomes: A Systematic Review and Meta-Analysis. Nutrients.

[12] Asif A, Farooq N (2023). StatPearls.

[13] Mashayekh-Amiri S, Mohammad-Alizadeh-Charandabi S, Abdolalipour S (2022). Myo-inositol supplementation for prevention of gestational diabetes mellitus in overweight and obese pregnant women: a systematic review and meta-analysis. Diabetol Metab Syndr.

[14] Motuhifonua SK, Lin L, Alsweiler J, Crawford TJ, Crowther CA (2023). Antenatal dietary supplementation with myo-inositol for preventing gestational diabetes. Cochrane Database of Systematic Reviews.

[15] Department of Health and Social Care (2019). Physical activity for adults and older adults: 19 and over.

[16] NHS (2023). Planning your pregnancy.

[17] Kamińska K, Stenclik D, Błażejewska W, Bogdanski P, Moszak M (2024). Probiotics in the Prevention and Treatment of Gestational Diabetes Mellitus (GDM): A Review. Nutrients.

[18] Davidson SJ, Barrett HL, Price SA, Callaway LK, Dekker Nitert M (2021). Probiotics for preventing gestational diabetes. Cochrane Database Syst Rev.

[19] NICE Diabetes in pregnancy: management from preconception to the postnatal period: NICE guideline [NG3].

[20] NHS (2022). Treatment -Polycystic ovary syndrome.

